# Development of a Japanese version of the SARC-F for diabetic patients: an examination of reliability and validity

**DOI:** 10.1007/s40520-016-0668-5

**Published:** 2016-11-10

**Authors:** Satoshi Ida, Kazuya Murata, Daiki Nakadachi, Yuki Ishihara, Kanako Imataka, Akihiro Uchida, Kou Monguchi, Ryutaro Kaneko, Ryoko Fujiwara, Hiroka Takahashi

**Affiliations:** 1 Department of Diabetes and Metabolism, Ise Red Cross Hospital, 1-471-2, Funae, 1-chome, Ise-shi, Mie 516-8512 Japan; 2 Department of Rehabilitation, Ise Red Cross Hospital, 1-471-2, Funae, 1-chome, Ise-shi, Mie 516-8512 Japan

**Keywords:** Sarcopenia, Simple screening, Reliability, Validity, Diabetes

## Abstract

**Background:**

SARC-F is a 5-item, self-administered questionnaire developed to screen sarcopenia. To date, no Japanese version of the SARC-F has been developed.

**Aims:**

To create a Japanese version of the SARC-F (SARC-F-J), a questionnaire for diabetic patients, and to investigate its reliability and validity.

**Methods:**

This was a cross-sectional study. A Japanese translation of the SARC-F was created and revised, and the authors of the original version of the SARC-F verified the back-translation. The questionnaire was tested in diabetic outpatients aged ≥65 years who had received treatment at our hospital. After 14 weeks, the kappa coefficient was used to evaluate the retest reliability. Using the diagnostic criteria for sarcopenia based on the European Working Group on Sarcopenia in Older People as the reference standard, we calculated the sensitivity, specificity, positive predictive value, and negative predictive value of the SARC-F-J.

**Results:**

The study comprised 207 patients (men, 60.8%; women, 39.2%). The kappa coefficient was 0.66. For men and women, the sensitivities were 14.6 and 33.3%, specificities were 85.8 and 72.4%, positive predictive values were 33.3 and 17.3%, and negative predictive values were 65.7 and 86.2%, respectively.

**Discussion:**

The probability of identifying the condition is considered high when patients are diagnosed with sarcopenia using SARC-F-J.

**Conclusions:**

The retest reliability of SARC-F-J was regarded to be good. When EWGSOP was assumed as a reference, the specificity of SARC-F-J was high. Because the sensitivity was low, patients with sarcopenia could not be screened, and hence, attention is needed.

## Background

Recently, the incidence rate of type 2 diabetes has been increasing and diabetes is indicated to be associated with an increased risk of cardiovascular events, reduced quality of life, and increased medical costs [1–4]. Therefore, antidiabetic measures are an important issue. Japan is a country with the most progressively aging population in the world, and its elderly population has been reported to have problems of reduced muscle mass and physical function [5, 6]. Although the international consensus on sarcopenia remains unsettled, a definition has been proposed by the European Working Group on Sarcopenia in Older People (EWGSOP) [7]. According to this definition, sarcopenia is diagnosed when a patient has (1) low muscle mass: two standard deviations (SDs) or less of the mean adult value (based on dual X-ray absorptiometry or bioelectrical impedance analysis), in addition to (2) low muscle strength: grip strength of ≤30 kg in men and ≤20 kg in women or (3) low physical activity: a walking speed of ≤0.8 m/s. Reduced muscle mass, grip strength, and walking speed have been reported to be associated with the onset of impaired activities of daily living (ADL), risk of falls, and death [8, 9].

The prevalence of sarcopenia is higher in diabetic patients than in non-diabetic individuals [10]. It is thought that muscle quantity and weakness decrease by inappropriate nourishment and exercise custom, low levels of sex and growth hormones, a decrease in the nerve fiber, insulin resistance, obesity, and participation of the inflammatory cytokine; this is the mechanism by which sarcopenia is promoted in a diabetic patient [10]. It is considered important to identify diabetic patients with sarcopenia and to treat them appropriately. However, the diagnosis of sarcopenia is complicated and often difficult to perform in routine care. Therefore, it is thought that it is necessary to diagnose a person at a risk of sarcopenia with precedence. Malmstrom and Morley [11] developed a questionnaire called SARC-F, which screens patients for sarcopenia on the basis of five questions and reported on its validity [12]. A SARC-F Japanese version for the general population and diabetic patients has, however, not been developed. Therefore, it is important that sarcopenia be easily screened in a diabetic patient. Therefore, we created a Japanese version of the SARC-F for Japanese diabetic patients called the “SARC-F-J” and investigated its reliability and validity.

## Methods

### Translation

The SARC-F is a tool that allows us to easily screen sarcopenia, and its usefulness has been appraised [11, 12]. The questionnaire is composed of five questions, covering strength, assistance in walking, rising from a chair, climbing stairs, and falls. Answers are given on the following three-point scale: “No difficulty at all,” “Some difficulty,” and “Extreme difficulty or inability.” Answers to the question on falls are given on the following three-point scale: “None at all,” “1–3 falls,” and “4 or more falls.” A score of 0, 1, or 2 points is given for each of the answers, respectively. The total score can be a minimum of 0 points or a maximum of 10 points. A diagnosis of sarcopenia is made with a total score of 4 or higher. The original SARC-F was translated into Japanese independently by two members of our group (SI and RK). The translated questionnaires were revised following a discussion of each item. In question item 1, an explanation “Two 2-L plastic bottles or one off-the-shelf bag of rice” was added as a specific example of 10 lb (≒4.5 kg). Next, we had a native English speaker back-translate the two Japanese translated questionnaires. The back-translated questionnaires were sent to the authors of the SARC-F to check that there were no discrepancies compared to the original version. Consequently, the authors of the SARC-F gave their consent to SARC-F-J (December 12, 2015).

### Study design and subjects

This was a cross-sectional study of diabetic patients visiting the Department of Diabetes and Metabolism, Ise Red Cross Hospital, Mie, Japan, on an outpatient basis. All patients gave their written informed consent to participate in this study, and the Ethical Review Board of the Ise Red Cross Hospital approved this study (December 2015). The following patients were included in the study: diabetic patients aged ≥65 who had visited our hospital on an outpatient basis between December 2015 and February 2016. The exclusion criteria were secondary diabetes, alcoholism, a diagnostic history of severe mental illness, patients with a pacemaker implanted, those undergoing bilateral knee and/or hip replacement surgery, those having home oxygen therapy, those diagnosed with cardiac insufficiency within the previous 6 months, and those unable to take the questionnaire by themselves [13].

### Evaluation using the questionnaires

The Tokyo Metropolitan Institute of Gerontology (TMIG) Index of Competence [14], the Japanese version of the Falls Efficacy Scale (FES) [15], and the Kaigo–Yobo Checklist (CL) [16] were used for evaluation. The investigators distributed the questionnaires to patients during outpatient visits, and the patients filled them out. The TMIG Index of Competence is widely used as a means of evaluating advanced ADL (AADL), whose reliability and validity have been verified [17]. This index is composed of 13 items, including questions on meal preparation, money management, use of transportation, intellectual activeness, and social role. The answers are either yes or no. The scores range from 0 to 15 points, with a higher score indicating AADL. The Japanese version of the FES is the original FES [18] modified by Haga et al. This scale examines 10 ADL items (bathing, opening cupboards and chests of drawers, dressing and undressing, sitting and standing, lying down on and getting up from a futon, walking around the house, performing simple household chores, basic shopping, basic cleaning, and quickly answering the phone) on a four-point scale regarding the level of confidence in being able to perform these activities without falling (1, no confidence at all; 2, not much confidence; 3, some confidence; 4, full confidence). A score range of 10–40 points and a higher score indicates stronger confidence. The CL is a scale developed in Japan to screen for elderly individuals who may be in need of nursing care in the future. This list is composed of groups of questions focusing on seclusion, falls, and malnutrition, which are important risk factors for requiring care. In total, there are 15 questions, and overall scores range from 0 to 15 points. A higher score has been suggested to indicate a higher likelihood to be in need of nursing care in the future, and the validity of this scale has also been tested [19].

### Measurement of muscle mass, grip strength, and physical function

We evaluated muscle mass, muscle strength, and walking speed, which need to be measured to diagnose sarcopenia. In the EWGSOP definition, sarcopenia is diagnosed when patients have reduced muscle mass in addition to reduced muscle strength or reduced muscle performances (assessed by walking speed or short physical performance battery test). Muscle mass (kg/m^2^) was measured as follows: the total appendicular skeletal muscle mass (ASM) was measured using multifrequency bioelectrical impedance analysis (InBody 230, Biospace, Korea), and then, the skeletal muscle mass index (SMI) was calculated [SMI (kg/m^2^) = ASM/height^2^]. Patients were considered to have low muscle mass when their SMI was ≤8.8 kg/m^2^ for men and ≤6.4 kg/m^2^ for women, which is equivalent to −2 SDs or lower of the mean value for young adults (18–40 years of age) according to the criteria for low muscle mass [20]. Grip strength was measured using a Smedley-type hand dynamometer (Grip-D, Takei Scientific Instruments Co., Ltd., Japan). First, while a patient was standing, half the length from the tip of the index finger to the base of the thumb was set as the reference position. Then, the second measurement was taken ≥30 s after the first measurement. The measurements were performed twice on both the right and left sides. We adopted the mean between the maximal values for each hand, with the unit set to kilogram [21]. Patients were considered to have low muscle strength when the results were ≤30 kg for men and ≤20 kg for women. To measure normal walking speed, patients were asked to walk an 11-m linear course at their usual speed. The walking speed was calculated by dividing the time it took patients to walk a 5-m section of this course by the number of seconds it took to complete the section (using the data from the 3 and 8 m marks) [22]. When the speed was ≤0.8 m/s, the patient was considered to have a reduced normal walking speed, i.e., low physical activity. When patients were incapable of walking, they were also considered to have low physical activity.

### Measurement of other variables

Age; gender; presence or absence of smoking and drinking habits; classification and duration of diabetes; use of antidiabetics; fasting serum C-peptide; fasting blood glucose; hemoglobin A1c (HbA1c); and the presence or absence of hypertension, dyslipidemia, diabetic retinopathy, diabetic neuropathy, diabetic nephropathy, and cardiovascular disease were described. The question regarding smoking habit was phrased as follows: “Do you currently smoke tobacco habitually?” and was answered with a yes or no. The question about drinking habit was phrased as follows: “Do you have a habit of drinking alcohol (rice wine, beer, distilled spirits, plum wine, etc.)?” and was also answered with a yes or no. Blood sampling data were collected using venous blood. Diabetes was classified as “type 1,” “type 2,” or “other” in the case of secondary diabetes, in accordance with the diagnostic criteria of the Japan Diabetes Society [23]. Serum C-peptide was measured using an enzyme-linked immunosorbent assay, and serum lipids were measured with a standard enzyme method. Plasma glucose and HbA1c levels were determined in accordance with the National Glycohemoglobin Standardization Program, using the glucose oxidase method and high-pressure liquid chromatography, respectively. Systolic and diastolic blood pressures were measured in an examination room, and patients were considered to have hypertension if they had systolic blood pressure ≥130 mmHg, diastolic blood pressure ≥80 mmHg, or were using oral antihypertensives [24]. Patients were considered to have dyslipidemia if they had triglycerides ≥150 mg/dL, high-density lipoprotein cholesterol <40 mg/dL, low-density lipoprotein cholesterol ≥120 mg/dL (≥100 mg/dL in the case of a coronary artery disease), or used oral hypolipidemic drugs [25]. Diabetic retinopathy was diagnosed by an ophthalmologist. Patients were considered to have diabetic neuropathy if any of the following were observed: reduced Achilles tendon reflex, reduced vibratory perception in the lateral malleolus, or abnormalities detected by a nerve conduction test. Patients were considered to have cardiovascular disease if they currently suffered from or had a history of ischemic heart disease such as angina pectoris or myocardial infarction, or cerebrovascular disease such as cerebral hemorrhage or infarction.

### Statistical analysis

Patient background, measurement items, and the score distribution on the SARC-F-J were recorded on the basis of gender and the presence or absence of sarcopenia according to the SARC-F-J. The SARC-F-J was re-administered 14 days after the first scoring, and the retest reliability was evaluated using the kappa coefficient. The presence or absence of sarcopenia according to the EWGSOP definition was also recorded on the basis of gender and the presence or absence of sarcopenia according to the SARC-F-J. The sensitivity, specificity, positive predictive value, and negative predictive value of the SARC-F-J were calculated using the diagnostic criteria for sarcopenia with the EWGSOP definition as the reference standard. To examine the validity of the SARC-F-J, Pearson correlation coefficients were determined between the SARC-F-J and TMIG Index of Competence, FES, and CL. Kappa coefficient and Pearson coefficients were interpreted as very good if 0.81–1.00, good if 0.61–0.80, moderate if 0.41–0.60, and poor to fair if <0.40 [26, 27]. The level of significance (two-tailed) was set at <0.05, and Stata version 12.0 (Stata Corporation LP, College Station, TX, USA) was used for the analysis.

## Results

### Characteristics of the analysis population

Two hundred and twenty-two patients who met the inclusion criteria were included in this study. Of these patients, 15 were excluded because of missing answers in the SARC-F-J or missing measurement items or other data necessary for a sarcopenia diagnosis as defined by EWGSOP. The remaining 207 patients were analyzed in the study. The patients’ backgrounds and distribution of SARC-F-J scores are presented in Tables 1 and 2, respectively. When the patients’ backgrounds were analyzed on the basis of the presence or absence of sarcopenia (no sarcopenia group vs. sarcopenia group) according to the SARC-F-J, the men’s mean ages were 71.6 ± 5.1 and 74.2 ± 7.0 years (*P* = 0.166), respectively, and the women’s mean ages were 71.6 ± 5.1 and 73.9 ± 6.5 years (*P* = 0.616), respectively. In men, HbA1c levels were 7.2 ± 1.0 and 7.2 ± 0.8% (*P* = 0.126), respectively, and in women they were 7.4 ± 0.9 and 7.1 ± 1.3% (*P* = 0.947), respectively. In addition, the duration of diabetes in men was 18.6 ± 11.6 and 15.0 ± 8.1 years (*P* = 0.521), respectively, and 17.3 ± 9.8 and 17.9 ± 9.8 years (*P* = 0.695) in women, respectively. The shared characteristics among both men and women in the sarcopenia and no sarcopenia groups were significantly lower BMI (*P* < 0.001 and *P* < 0.001, respectively). The sarcopenia group also tended to have a lower rate of oral drug use and higher rates of insulin use. This group additionally tended to have lower grip strength.

**Table 1:** Characteristics of the analysis population according to EWGSOP definition of sarcopenia

	Men			Women		
	No sarcopenia *n* = 108	Sarcopenia *n* = 18	*P*	No sarcopenia *n* = 58	Sarcopenia *n* = 23	*P*
Age (years), mean (SD)	71.5 (5.3)	73.0 (5.8)	0.166	72.3 (5.7)	71.5 (5.1)	0.616
BMI (kg/m^2^), mean (SD)	25.0 (3.3)	21.2 (4.1)	0.001	24.4 (4.0)	19.9 (2.6)	<0.001
T1DM/T2DM/other (%)	2.3/96.4/1.3	0/100/0	0.598	12.3/87.7/0	8.7/82.6/8.7	0.776
HbA1c (%), mean (SD)	7.3 (1.0)	7.0 (0.9)	0.126	7.3 (1.0)	7.3 (1.2)	0.947
Duration of diabetes (years), mean (SD)	17.5 (10.8)	19.0 (12.1)	0.521	17.7 (8.7)	16.4 (15.1)	0.695
Alcohol consumption (%)	17.6	12.1	0.433	5.7	4.4	0.392
Smoking (%)	23.5	34.1	0.208	5.7	4.1	0.392
Hypertension (%)	83.3	63.4	0.013	72.4	66.6	0.681
Dyslipidemia (%)	79.7	55.0	0.004	71.6	54.5	0.255
Retinopathy (%)	31.7	34.1	0.789	34.7	33.3	0.922
Neuropathy (%)	29.4	43.9	0.108	36.2	41.6	0.719
Nephropathy (%)	52.0	55.0	0.759	53.1	55.5	0.891
Cardiovascular diseases (%)	20.0	21.9	0.800	18.8	8.3	0.374
Oral hypoglycemic agents (%)	82.3	65.8	0.039	81.1	66.6	0.255
GLP-1 analog (%)	5.8	4.8	0.818	18.8	8.3	0.374
Insulin (%)	55.9	63.4	0.427	69.5	66.6	0.841
TMIG Index of Competence scores, mean (SD)	11.6 (1.6)	11.1 (2.7)	0.185	11.5 (3.1)	11.1 (1.6)	0.673
FES scores, mean (SD)	36.2 (4.6)	35.2 (4.7)	0.274	35.0 (5.1)	32.8 (10.4)	0.261
CL scores, mean (SD)	2.4 (2.2)	2.9 (2.3)	0.293	2.8 (2.3)	3.4 (1.8)	0.392
SMI (kg/m^2^), mean (SD)	9.3 (0.9)	7.7 (0.6)	<0.001	7.4 (1.0)	6.0 (0.2)	<0.001
Grip strength (kg), mean (SD)	31.5 (6.2)	25.9 (4.4)	<0.001	19.3 (4.4)	18.0 (3.2)	0.376
Walking speed (m/s), mean (SD)	1.04 (0.2)	1.02 (0.2)	0.566	0.96 (0.2)	0.93 (0.2)	0.707

Cardiovascular diseases included angina pectoris, myocardial infarction, and stroke

*SD* Standard deviation, *BMI* body mass index, *T1DM/T2DM* type 1/type 2 diabetes mellitus, *HbA1c* hemoglobin A1c, *GLP-1* glucagon-like peptide-1, *TMIG* Tokyo Metropolitan Institute of Gerontology, *FES* Falls Efficacy Scale, *CL* Kaigo–Yobo Checklist, *SMI* skeletal muscle mass index

**Table 2:** Distribution of SARC-F-J score

	Men		Women	
	SARC-F-J, frequency (%)		SARC-F-J, frequency (%)	
	No sarcopenia *n* = 108	Sarcopenia *n* = 18	No sarcopenia *n* = 58	Sarcopenia *n* = 23
*SARC-F-J*				
1. Strength				
No difficulty	95 (87.9)	1 (5.6)	45 (77.6)	1 (4.3)
Some difficulty	12 (11.2)	11 (61.1)	13 (22.4)	14 (60.9)
Extreme difficulty or inability	1 (0.9)	6 (33.3)	0 (0)	8 (34.8)
2. Assistance in walking				
No difficulty	105 (97.2)	5 (27.8)	56 (96.6)	8 (34.8)
Some difficulty	3 (2.8)	10 (55.6)	2 (3.4)	14 (60.9)
Extreme difficulty or inability	0 (0)	3 (16.6)	0 (0)	1 (4.3)
3. Rise from a chair				
No difficulty	95 (88.0)	1 (5.6)	51 (87.9)	4 (17.4)
Some difficulty	13 (12.0)	14 (77.8)	7 (12.1)	15 (65.2)
Extreme difficulty or inability	0 (0)	3 (16.6)	0 (0)	4 (17.4)
4. Climb stairs				
No difficulty	71 (65.7)	1 (5.6)	37 (63.8)	0 (0)
Some difficulty	37 (34.3)	10 (55.6)	19 (32.8)	14 (60.9)
Extreme difficulty or inability	0 (0)	7 (38.8)	2 (3.4)	9 (39.1)
5. Falls				
None	92 (85.2)	6 (33.3)	41 (70.7)	12 (52.1)
1–3	15 (13.9)	8 (44.5)	17 (29.3)	10 (43.5)
>4	1 (0.9)	4 (22.2)	0 (0)	1 (4.4)

### Reliability

The kappa coefficient evaluation index of retest reliability for the SARC-F-J was 0.66.

### SARC-F-J and EWGSOP definitions of sarcopenia

The distributions of the presence or absence of sarcopenia according to the EWGSOP and SARC-F-J definitions are presented on the basis of gender in Table 3. The numbers of men and women with sarcopenia according to the EWGSOP criteria were 41 (32.5%) and 12 (14.8%), respectively.

**Table 3:** SARC-F-J and EWGSOP definitions of sarcopenia

	Men		Women	
	SARC-F-J, frequency (%)/mean (SD)		SARC-F-J, frequency (%)/mean (SD)	
	No sarcopenia *n* **=** 108	Sarcopenia *n* **=** 18	No sarcopenia *n* **=** 58	Sarcopenia *n* **=** 23
EWGSOP criteria				
No sarcopenia	73 (67.6)	12 (66.7)	50 (86.2)	19 (82.6)
Sarcopenia	35 (32.4)	6 (33.3)	8 (13.8)	4 (17.4)

*SARC-F-J* a Japanese version of SARC-F, *EWGSOP* European Working Group on Sarcopenia in Older People

### Sensitivity, specificity, positive predictive value, and negative predictive value of the SARC-F-J

The sensitivities, specificities, positive predictive values, and negative predictive values of the SARC-F-J determined using the EWGSOP definition of sarcopenia as the reference standard are presented in Table 4. For men and women, the sensitivities were 14.6 and 33.3%, the specificities were 85.8 and 72.4%, the positive predictive values were 33.3 and 17.3%, and the negative predictive values were 65.7 and 86.2%, respectively.

**Table 4:** Sensitivity, specificity, positive predictive value, and negative predictive value of SARC-F-J

Sensitivity, %	Specificity, %		Positive predictive value, %	Negative predictive value, %
Men	14.6	85.8	33.3	67.5
Women	33.3	72.4	17.3	86.2

### Association between TMIG Index of Competence, FES, and CL scores, and SARC-F-J-defined sarcopenia

The Pearson correlation coefficients between the SARC-F-J and the TMIG Index of Competence, FES, and CL are presented in Table 5. For men and women, the correlation coefficients were −0.363 (*P* < 0.001) and −0.468 (*P* < 0.001) for TMIG Index of Competence, −0.560 (*P* < 0.001) and −0.535 (*P* < 0.001) for FES, and 0.591 (*P* < 0.001) and 0.657 (*P* < 0.001) for CL, respectively.

**Table 5:** Correlation between SARC-F-J and TMIG, FES, and CL

	Men *n* **=** 126		Women *n* **=** 81	
	Pearson’s correlation coefficient	*P*	Pearson’s correlation coefficient	*P*
TMIG Index of Competence scores	−0.363	<0.001	−0.468	<0.001
FES scores	−0.560	<0.001	−0.535	<0.001
CL scores	0.591	<0.001	0.657	<0.001

*TMIG* Tokyo Metropolitan Institute of Gerontology, *FES* Falls Efficacy Scale, *CL* Kaigo–Yobo Checklist

## Discussion

In the present study, a Japanese version of the SARC-F (SARC-F-J) for diabetics was created after obtaining the approval from the authors of the original version of the SARC-F. We then verified the retest reliability and validity of the SARC-F-J. Using the EWGSOP definition for sarcopenia as the reference standard, we obtained low values for both men and women for SARC-F-J sensitivity (men 14.6%, women 33.3%) and positive predictive values (men 33.3%, women 17.3%). On the other hand, we obtained relatively high values for both men and women for SARC-F-J specificity (men 85.8%, women 72.4%) and for the negative predictive values (men 67.5%, women 86.2%). The probability of identifying sarcopenia is considered high when patients are diagnosed as having sarcopenia using SARC-F-J. Moreover, the probability of being able to rule out sarcopenia is also considered high when patients are diagnosed as not having sarcopenia using the SARC-F-J at facilities with a pretest probability similar to our facility.

The sensitivity and specificity of the SARC-F-J were compared with the data from an earlier study. In a study conducted on Chinese people, the sensitivity and specificity of the SARC-F for men were 4.2 and 98.7%, respectively, and for women they were 9.9 and 94.4%, respectively [12]. These results differed from those obtained in the present study, probably because of differences in question interpretation (nuance) arising from racial, social, and cultural differences between the translated and original versions of the SARC-F. Furthermore, the subjects in the aforementioned earlier study were members of the general population, whereas the subjects in the present study were diabetic patients, which may have influenced the results. In other words, diabetic patients have been identified as being more prone to suffering from sarcopenia than non-diabetic individuals [10]. Compared with the aforementioned earlier study, the positive predictive value of the SARC-F-J in the present study was higher, whereas the negative predictive value was lower. This may have been due to the high prevalence of sarcopenia at our facility, i.e., a high pretest probability. Moreover, the sample size in the earlier study was larger than that in the present study, which might have also affected the results. The retest reliability of the SARC-F-J scored a kappa coefficient of 0.66. Because the earlier study did not verify the retest reliability of the SARC-F, its results cannot be directly compared with those of the present study; however, reproducibility is generally good with kappa coefficients of ≥0.6 [26]. The retest reliability was, therefore, considered reasonable.

Although the correlation was poor in men, a moderate correlation between SARC-F-J and TMIG was observed in women. In addition, a moderate correlation between SARC-F-J and FES was observed in both men and women. A correlation that was more than moderate between SARC-F-J and CL was observed in both men and women. An earlier study reported a correlation between the presence of sarcopenia according to the SARC-F and reduced physical function, instrumental ADL, and FES score [28]. The TMIG Index of Competence and CL are questionnaires that measure pathologies known to be closely related to sarcopenia, such as reduced instrumental ADL and frailty [29, 30]. Sarcopenia is also known to be associated with fall risk [31]. The questions in the SARC-F-J in the present study may be able to test the disease concept of sarcopenia to a certain degree, i.e., provide results that support its validity.

The present study is the first to verify the reliability and validity of sarcopenia screening with a Japanese version of the SARC-F for diabetic patients. Because this questionnaire can identify patients at a high risk of sarcopenia, it may aid in reducing medical costs and time needed for the evaluation of muscle mass, physical function, and other factors considered necessary for subsequent definitive diagnosis. Moreover, sarcopenia has been suggested to be linked to falls, reduced instrumental ADL, and outcomes such as cardiovascular events [8]. Some studies have reported the possibility that therapeutic interventions such as resistance exercise and protein diets could prevent fractures and loss of muscle mass and strength in cases of sarcopenia [32–34]. This suggests the importance of screening sarcopenia at an early stage and administering appropriate care. The SARC-F-J may serve as a useful screening tool in such cases.

The present study, however, had several limitations. The first was the bias in patient characteristics because many patients had diabetes and poor glycemic control for a long period of time. Moreover, our hospital employs diabetes specialists and has a comparatively large number of patients with severe disease. Caution will, therefore, be needed when applying the SARC-F-J to diabetic patients in other settings such as primary care. Studies of patients with relatively mild diabetes, those with short disease durations, and diabetic patients in primary care settings are warranted. The second limitation was the cutoff used for screening sarcopenia. It is reported that the prevalence of sarcopenia varies according to a difference in the cutoff for SMI, grip strength, walking speed, and the measuring equipment used [35, 36]. Therefore, the cutoffs may have influenced the results of this study and the prevalence of sarcopenia may be different than what was expected. Further examinations using a different cutoff or different measuring equipment are necessary in the future. The third limitation was that because this was a cross-sectional study, changes in the prognoses of patients diagnosed with sarcopenia using the SARC-F-J or changes in SARC-F-J scores following therapeutic intervention were not investigated. Patient prognoses will need to be evaluated and the responsiveness of SARC-F-J scores will need to be verified in future longitudinal and interventional studies, respectively. The fourth limitation was that there was no evaluation of cognitive function in the present study. Despite obtaining results that guaranteed reliability to a certain extent, our findings suggested that reduced cognitive function may have affected the validity of the responses to the questionnaire. Finally, because the present study had a comparatively small sample size, it is possible that the evaluation of screening performance was inadequate. Verification using a larger sample size will be needed in the future.

In conclusion, in a Japanese diabetic patient, the retest reliability of SARC-F-J was regarded to be good. When we assumed EWGSOP as a standard reference, the specificity of SARC-F-J was high. Because the sensitivity was low, patients with sarcopenia could not be screened, and hence, attention is needed.
