# Correction to: Development of a Japanese version of the SARC-F for diabetic patients: an examination of reliability and validity

**DOI:** 10.1007/s40520-019-01308-1

**Published:** 2019-08-19

**Authors:** Satoshi Ida, Kazuya Murata, Daiki Nakadachi, Yuki Ishihara, Kanako Imataka, Akihiro Uchida, Kou Monguchi, Ryutaro Kaneko, Ryoko Fujiwara, Hiroka Takahashi

**Affiliations:** 1 Department of Diabetes and Metabolism, Ise Red Cross Hospital, 1-471-2, Funae, 1-chome, Ise-shi, Mie 516-8512 Japan; 2 Department of Rehabilitation, Ise Red Cross Hospital, 1-471-2, Funae, 1-chome, Ise-shi, Mie 516-8512 Japan

## Correction to: Aging Clin Exp Res (2017) 29:935–942 10.1007/s40520-016-0668-5

The article Development of a Japanese version of the SARC-F for diabetic patients: an examination of reliability and validity, written by Satoshi Ida, Kazuya Murata, Daiki Nakadachi, Yuki Ishihara, Kanako Imataka, Akihiro Uchida, Kou Monguchi, Ryutaro Kaneko, Ryoko Fujiwara and Hiroka Takahashi was originally published electronically on the publisher’s internet portal (currently SpringerLink) on 10 November 2016 without open access.

With the author(s)’ decision to opt for Open Choice the copyright of the article changed on 15 August 2019 to © The Author(s) 2019 and the article is forthwith distributed under the terms of the Creative Commons Attribution 4.0 International License (http://creativecommons.org/licenses/by/4.0/), which permits use, duplication, adaptation, distribution and reproduction in any medium or format, as long as you give appropriate credit to the original author(s) and the source, provide a link to the Creative Commons license and indicate if changes were made. The original article has been corrected.

